# Needs-oriented discharge planning and monitoring for high utilisers of psychiatric services (NODPAM): Design and methods

**DOI:** 10.1186/1472-6963-8-152

**Published:** 2008-07-21

**Authors:** Bernd Puschner, Sabine Steffen, Wolfgang Gaebel, Harald Freyberger, Helmfried E Klein, Tilman Steinert, Rainer Muche, Thomas Becker

**Affiliations:** 1 Department of Psychiatry II, Ulm University, BKH Günzburg, Ludwig-Heilmeyer-Str. 2, 89312 Günzburg, Germany; 2 Department of Psychiatry and Psychotherapy, Heinrich-Heine-University Düsseldorf, Bergische Landstr. 2, 40629 Düsseldorf, Germany; 3 Department of Psychiatry and Psychotherapy, Greifswald University, Rostocker Chaussee 70, 18437 Stralsund, Germany; 4 Department of Psychiatry and Psychotherapy, Regensburg University, Universitätsstr. 84, 93053 Regensburg, Germany; 5 Department of Psychiatry I, Ulm University, Ravensburg, Weingartshofer Str. 2, 88214 Ravensburg, Germany; 6 Institute for Biometrics, Ulm University, Schwabstr. 13, 89075 Ulm, Germany

## Abstract

**Background:**

Attempts to reduce high utilisation of psychiatric inpatient care by targeting the critical time of hospital discharge have been rare.

**Methods:**

This paper presents design and methods of the study "Effectiveness and Cost-Effectiveness of Needs-Oriented Discharge Planning and Monitoring for High Utilisers of Psychiatric Services" (NODPAM), a multicentre RCT conducted in five psychiatric hospitals in Germany. Inclusion criteria are receipt of inpatient psychiatric care, adult age, diagnosis of schizophrenia or affective disorder, defined high utilisation of psychiatric care during two years prior to the current admission, and given informed consent. Consecutive recruitment started in April 2006. Since then, during a period of 18 months, comprehensive outcome data of 490 participants is being collected at baseline and during three follow-up measurement points.

The manualised intervention applies principles of needs-led care and focuses on the inpatient-outpatient transition. A trained intervention worker provides two intervention sessions: (a) Discharge planning: Just before discharge with the patient and responsible clinician at the inpatient service; (b) Monitoring: Three months after discharge with the patient and outpatient clinician. A written treatment plan is signed by all participants after each session.

Primary endpoints are whether participants in the intervention group will show fewer hospital days and readmissions to hospital. Secondary endpoints are better compliance with aftercare, better clinical outcome and quality of life, as well as cost-effectiveness and cost-utility.

**Discussion:**

If a needs-oriented discharge planning and monitoring proves to be successful in this RCT, a tool will be at hand to improve patient outcome and reduce costs via harmonising fragmented mental health service provision.

**Trial Registration:**

ISRCTN59603527

## Background

Large numbers of psychic patients do not receive aftercare in the community during the period immediately following hospital discharge. The average rate of utilisation of aftercare is about 50%, with a wide range between 22 and 90% depending on the definition of aftercare [1-3]. More specifically, one third to one half of patients with schizophrenia and related disorders miss their first scheduled outpatient appointment after discharge [4]. In addition, for those who receive follow-up, the delay between discharge and receipt of aftercare (operationalised as first outpatient visit) has been found to be substantial [1,5]. Thus, limited continuity of service provision is pervasive, and time lags arising in this process have been found to increase the probability of relapse and to negatively affect patients' quality of life [1].

There have been some research efforts aimed at identifying the causes for this failure to utilise psychiatric services (for an overview see [2]). Results based on patient variables have been mostly negative, while service system variables, e.g. availability of discharge planning, have been found to facilitate access to aftercare. There is some evidence suggesting that patients who receive discharge planning are more likely to utilise outpatient mental health services and are less prone to become socially isolated and require rehospitalisation. Thus, in a sample of N = 104 inpatients with schizophrenia or schizoaffective disorder, Olfson et al. [6] found that those who had received discharge planning showed significantly better aftercare compliance (98.1 vs. 62.7%), fewer rehospitalisations (40% reduction), and better clinical outcome (34.5 vs. 41.0 BPRS-points, ES = .57) at three-month follow-up. Furthermore, in a sample of 229 inpatients with a primary psychiatric diagnosis, Boyer et al. [1] found that patients were significantly more likely to keep their initial outpatient appointment if they were involved in the outpatient programme before discharge (OR = 2.14 – 3.89) or if the discharge plan was discussed between inpatient staff and outpatient clinicians (OR = 3.17).

It appears that rather simple measures – such as timely face-to-face contact of the inpatient with the follow-up outpatient therapist(s) and/or smooth transition between in- and outpatient treatment – are likely to bring about an increase in successful community tenure. Also a time-limited critical time intervention [6,7] has been shown to yield clinical improvement. The impact of extensive strategies such as case management or the introduction of discharge coordinators, on the other hand, has been limited or controversial.

Nonetheless, the use of bridging strategies in routine practice is idiosyncratic at best. Boyer et al. [1] showed that while discussions between in- and outpatient teams regarding the arrangement of aftercare occur rather frequently (in two thirds of the cases), patient involvement with outpatient programmes while they are still in hospital is less common.

Since the 1980s considerable research effort has been devoted to high utilisation (HU) of psychiatric services. According to Hadley et al. [8], "the concept of 'heavy use' typically applies to those persons whose frequency of admission or duration of inpatient service is substantially beyond that of the majority of persons receiving similar treatment" (p. 280). Two extensive literature reviews on the topic (Kent et al. [9]: 200 publications identified, 72 described; Roick et al. [10]: 250 publications identified, 105 described) showed that overall, 10 – 30% of psychiatric patients are identified as high utilisers who consume between 50 and 80% of service resources.

Probably due to large differences between psychiatric service systems, there is no consensus on what constitutes HU. In operationalising HU, most authors solely relied on an arbitrary number of readmissions during a given time period – usually study duration. An analysis of nine studies pertaining to psychiatric inpatient treatment (cf. table 1 in Kent et al. [9]) showed that a mean number of 3.25 (SD = 1.75) admissions during 2.38 (SD = 1.51) years, i.e. 1.37 admissions per year, was used as the criterion for HU. This corresponds to Roick et al. [10] who found that overall, patients with 1 – 3 inpatient stays during one year are identified as a high utilisers. This measure is easily accessible, but has also been criticised (e.g. [11]) since patients with multiple short hospital stays might be wrongly included (since they do not use excessive resources), and patients with few very long hospital stays might be wrongly excluded, especially when observation time is short. Thus, some authors used cumulated length of stay (LOS) during a given period (in addition to or instead of number of readmissions) to identify HU. E.g. Lucas et al. [12] developed a "heaviness of use" score by combining the frequency of admissions with the total number of bed days. Exceptionally, direct treatment costs beyond cumulated inpatient costs including costs for non-inpatient care (day hospital treatment, outpatient and complementary care) was applied [13], but data collection is laborious and might not be worthwhile since costs for inpatient care make up the largest share (75–92% [9,14]) of total treatment costs.

**Table 1:** Study instruments by raters and measurement points.

	*T0*	*T1*	*T2*	*T3*
*Research worker*	CAN-EU	CAN-EU	CAN-EU	CAN-EU
	CSSRI-EU	CSSRI-EU	CSSRI-EU	CSSRI-EU
	BPRS	BPRS	BPRS	BPRS
	HAM-D	HAM-D	HAM-D	HAM-D
	MANSA	MANSA	MANSA	MANSA
	EQ-5D	EQ-5D	EQ-5D	EQ-5D
*Clinician*	GAF	GAF		
	CGI	CGI		
	STAR-C	STAR-C		
		ZUF-THERA		
		DP satisfaction		
*Patient*	SCL-90-R	SCL-90-R	SCL-90-R	SCL-90-R
	STAR-P	STAR-P	STAR-P	STAR-P
		ZUF-8		
		DP satisfaction		

*Notes*: DP = Discharge Planning. See text for other abbreviations.

### Objectives and purpose of the study

Objective of the study is to test the following hypotheses.

#### Primary

High utilisers of psychiatric services who receive a needs-oriented discharge planning and monitoring programme will show fewer hospital days and readmissions to hospital.

#### Secondary

Subjects receiving the intervention will show better compliance with aftercare as well as better clinical outcome and quality of life. Furthermore, the intervention will show cost-effectiveness and cost-utility, and community-based psychiatrists whose patients receive the new discharge protocol will show better compliance with treatment recommendations.

## Methods and design

A clinical trial entitled "Effectiveness and Cost-Effectiveness of Needs-Oriented Discharge Planning and Monitoring for High Utilisers of Psychiatric Services" (NODPAM) will be conducted. Coordinating centre (CC) is Ulm University's Department of Psychiatry II (Günzburg). Participating centres are the Departments of Psychiatry and Psychotherapy at the Universities of Düsseldorf, Greifswald, and Regensburg, and Ulm University's Department of Psychiatry I (Ravensburg). This paper describes design and methods of the NODPAM trial as outlined in the trial protocol which has been submitted to the funding body (German Research Foundation) on July 15, 2005 after having attended to the funders' and independent reviewers' comments on the original proposal and its revision. Before the start of recruitment in April 2006, minor changes have been applied^1^.

### Overview

**Figure 1:**
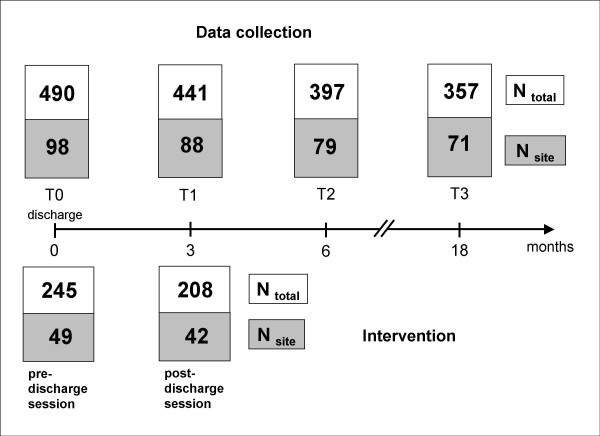
Trial design (timing and projected N for data collection and intervention).

NODPAM is a randomised controlled prospective trial with four measurement points during an 18-month study period (see Figure 1, upper part). Assessments will take place at baseline, three, six, and 18 months after discharge. We believe that this design is appropriate to answer the research questions since on one hand it allows to examine the short-term (three months after baseline), mid-term (six months) and long-term (18 months) effects of the intervention, and on the other hand, the burden on participants (patients and clinicians) is not too high which will help to keep low study attrition. In the event of rehospitalisation, data assessment will proceed as originally scheduled, allowing some delay in case the patient should be in an acute crisis making assessment impossible. Assessment will be carried out by a research worker (different in person from the intervention worker).

### Randomisation

A central randomisation procedure will be conducted by an independent unit (Ulm University's Institute for Biometrics). Stratification will be applied since we will conduct a multicentre trial, and, furthermore, since the intervention might have different effects as to diagnostic criteria, gender, and chronicity. In order to ensure an adequate distribution of patients with regard to these aspects, strata will be centre (five centres), primary diagnosis (ICD-10 Chapter V codes F20 – F29 vs. F30 – F39), gender (male vs. female) and chronicity (shorter vs. equal to or longer than three years). If during recruitment a patient fulfils inclusion criteria and has given informed consent, a fax will be sent to the randomisation centre which will perform randomisation, generate a patient code, and send it back to the study centre.

Cluster randomisation (at the clinician level) will not be employed since it would severely disrupt clinical routine and might result in undesired between-centre effects. Furthermore, in many wards of the participating services, only one clinician is present. Moreover, cross-contamination among inpatient clinicians is not to be expected since the intervention is low profile, restricted to one pre-discharge session, and will be carried out by a research worker with limited clinician involvement.

### Blinding

Due to the nature of the intervention, patients, clinicians, and intervention workers cannot be blinded to patient allocation. The natural flow of the study will also prevent the research worker from being blinded. Furthermore, blinding to patient allocation and centre affiliation of principal and responsible investigators as well as of the person performing randomisation is considered neither feasible nor necessary because no related bias is expected, and a blinded intermediate risk assessment, e.g. in order to identify excess mortality of participants as could be the case with invasive interventions or acutely critical conditions, is not required.

### Inclusion and exclusion criteria

#### Inclusion criteria

Psychiatric inpatients between 18–65 years with a primary diagnosis of schizophrenia, bipolar affective disorder, or major depression (ICD-10 Chapter V codes F20 – F29 or F30 – F39). Furthermore, subjects have to be identified as high utilisers of psychiatric inpatient care. In order to avoid misclassification, a two-fold criterion consisting of number of hospitalisations and cumulative LOS prior to the index admission will be applied; a subject will be included for participation if he or she, during a 24-month-period prior to admission: a) has been psychiatrically hospitalised at least twice with a cumulative LOS exceeding 30 days or b) has been psychiatric hospitalised at least once with a cumulative psychiatric LOS of more than 50 days. This criterion is in accordance with existing evidence and is also more comprehensive than the one suggested for psychiatric inpatient care in Germany by Roick et al. [15] who, regardless of LOS, categorised patients as high utilisers who, during a 30-month period, had had more than three admissions.

#### Exclusion criteria

(a) Primary diagnosis of substance abuse; (b) Presence of moderate or severe mental handicap (learning disability) or organic mental disorder; (c) Current treatment by forensic psychiatric services; (d) Insufficient command of the German language in order to receive the intervention, take part in the assessment interviews, and complete patient questionnaires; (e) Lacking capacity to give valid consent to participate; (f) Foreseeable inpatient or day care mental health treatment (including rehabilitation) extending seven days after discharge from psychiatric inpatient treatment.

### Outcome measures

Assessment will take place at baseline and three (3, 6 and 18 months) follow-up measurement points in each participating centre (see Figure 1). Patients will receive a remuneration of 30 € for each assessment. Secondary outcomes will be provided by independent raters (research workers trained in study instrument use), clinicians, and patients. See Table 1 for an overview of the instruments used.

#### Rater perspective (T0 – T3)

Cumulated *LOS *and number of *readmissions *during the study period (primary outcomes) will be assessed by a research worker at T1 – T3 via the German version of the "Client Sociodemographic and Service Receipt Inventory" (CSSRI-EU). This is a standardised instrument for the comprehensive assessment of mental health service use and costs [16] of which a German version is available [17]. It provides detailed information on direct (e.g. hospital in-patient days, out-patient/day care attendances, community-based service contacts, medication profile) and indirect (e.g. days of work loss, state benefits, source/level of income) costs and has been used in a number of German studies^2^.

Assessment of *needs *will be carried out with the German version of the "Camberwell Assessment of Need – European Version" (CAN-EU [18]), an interviewer-administered instrument comprising 22 individual domains of need. Staff and user ratings can be obtained; we administered only the user rating. *Psychopathology *will be assessed using the "Brief Psychiatric Rating Scale" (BPRS [19]) which is a standardised instrument for the assessment of psychopathological symptoms. *Depression *will be tapped into using the "Hamilton Depression Scale" (HAM-D [20]). *Quality of life *will be measured via a) the "Manchester Short Assessment of Quality of Life" (MANSA [21]) which is a disease specific instrument for the multidimensional assessment of objective and subjective quality of life in persons with severe mental illness and b) the EuroQol group's EQ-5D, a generic quality of life assessment instrument which has been developed for the computation of quality adjusted life years (QALYs) and whose psychometric properties and applicability in persons with severe mental illness have been tested [22].

#### Clinician perspective (T0 [inpatient clinician] and T1 [outpatient clinician])

*Psychosocial functioning *will be rated via the "Global Assessment of Functioning Scale" (GAF [23]) which has been shown to be a practical and valid instrument in psychiatric research [24]. Overall *psychological impairment *will be tapped into via the "Clinical Global Impression Scale" (CGI [25]). *Therapeutic relationship *(clinicians' point of view) will be assessed using a retranslated German version of the "Scale to assess the therapeutic relationship in community mental health care" clinician scale (STAR-C [26]).

*Satisfaction with therapeutic work in the ambulatory setting *(T1 only) will be covered with a self-developed scale (ZUF-THERA [27]) consisting of six rephrased items of the ZUF-8 [28], the German version of the "Client Satisfaction Questionnaire" (CSQ [29]). The items were rephrased in that way that outpatient clinicians could give an evaluation of the quality of the given treatment. *Satisfaction with discharge planning *(T1 and intervention group only) will be assessed with a self-developed nine item rating scale.

#### Patient perspective (T0 – T3)

*Psychological impairment *will be measured using the German version [30] of Derogatis' [31] "Symptom-Check-List" (SCL-90-R), a widely used and validated self-report scale. *Therapeutic relationship *(patients' point of view) will be assessed using the "Scale to assess the therapeutic relationship in community mental health care" patient scale (STAR-P [26]). *Satisfaction with outpatient treatment *(T1 only) will be covered for the outpatient setting with the German version (ZUF-8 [28]) of the "Client Satisfaction Questionnaire" (CSQ [29]). *Satisfaction with discharge planning *(T1 and intervention group only) will be assessed with a self-developed eight item rating scale.

### Sample size

Power calculation for a panel study with four points of assessment was based on the approach suggested by Hedeker et al. [32]. For a high utiliser population, previous research [33] found mean number of inpatient days during 12 months after discharge to be 47 (SD = 83) days (projected mean number for 18 months = 71 days). Based on existing studies, the mean reduction of inpatient days due to the planned intervention was assumed to be 40%. A small effect size (0.2 SD) should be detected with a power of 0.80 at a two-tailed significance level of 0.05. Panel attrition was estimated 10% at each measurement point. With regard to data analysis, a constant group effect over time with a random-effect structure and auto-correlated results was expected. A baseline sample size of 242 participants in each group was calculated to be sufficient.

Accordingly, after rounding to no decimals, a total sample size of 490 participants (N = 98 at each site) will be included in the study. Participants will be randomly assigned to the intervention (N = 245, N = 49 per site) or control group (N = 245, N = 49 per site).

### Intervention

For the intervention group, an intervention worker will provide a coherent package of needs-oriented discharge planning and monitoring focusing on the care process. The NODPAM intervention will apply principles of needs-led care (e.g. [34]) and focus on the inpatient-outpatient transition, with the intervention worker emphasising continuity of the care process vis-à-vis both patient and clinicians (e.g. [7]). The intervention worker will provide (and patients will be actively involved in) two manualised intervention sessions (each of about 45 minutes duration, see Figure 1, lower part):

#### Pre-discharge intervention session

The intervention worker obtains the relevant information of patients' met and unmet needs in a basic plan (results of the CAN rating) from the research worker. The first joint (pre-discharge) intervention session in general takes place seven days prior to hospital discharge with the following people participating: in-patient clinician, patient and carers if the patient wants to. There is a guided discussion of the critical domains of need identified on the basis of CAN ratings which require both current and post-discharge measures and/or interventions. The handwritten draft of the post-discharge treatment plan contains every addressed need with a precise problem definition, objectives, time-frame regarding goal attainment and persons responsible for the implementation. The treatment plan is signed by all participants. A typed version of the pre-discharge treatment plan will be sent to the outpatient clinician and the patient, and the accompanying letter will include the advice to discuss the plan and monitor its progress during every contact between them. The inpatient clinician is remunerated with CME credit points for each NODAPM intervention session and with a book voucher worth 50 € (once).

#### Post-discharge intervention session

Three months after discharge, the intervention worker arranges a second (post-discharge) intervention session including all stakeholders involved in the outpatient treatment phase: outpatient clinician, patient and relatives if the patient wants them to be present. Initially, a written protocol is drafted to summarise critical domains of need from the first session, their development since discharge, and the current CAN ratings (at 3-month follow-up). Participants are asked if they can name reasons for change (improvement or deterioration) or stability of ratings in the related domains.

Subsequently, a revised version of the pre-discharge treatment plan is developed on the basis of current met and unmet needs and signed by all participants. A typed version of the plan will be sent to the outpatient clinician and the patient with the advice that the plan should be continuously integrated in outpatient treatment, i. e. discussed and monitored during every contact between the outpatient clinician and patient for a period of at least three months. The outpatient clinician in private practice is remunerated with CME credit points and 100 € for each NODPAM intervention session.

### Statistical analysis

The primary endpoints will be derived from the difference in LOS and number of hospitalisations between T0 and T1, T2, and T3. The secondary endpoints will be calculated from the differences in sum (or – if applicable – subscale) scores of the secondary outcome measures between T0 and T1, T2, and T3. Multiple measurement points have been chosen in order to scrutinise the intervention's effect over time, especially the question of whether short- and mid-term effects would persist.

Analyses will start once baseline data have been collected and cleaned. Descriptives of all outcome measures will be produced, and outcome trajectories from T0 – T3 will be examined via exploratory analyses. The effect of the intervention on reduction of number of inpatient days as well as on clinical outcomes, quality of life, and compliance will be tested by means of random-effect regression models including a constant group effect across time [35-37]. Due to the application of a generalised least square (GLS) estimation weighted for the lengths of the time series for each case, these models allow the inclusion of cases with incomplete (unbalanced) data across panels. Due to expected skewness of the primary dependent variable (LOS), bootstrapping methods will be applied for the estimation of standard errors and confidence intervals. Cost-effectiveness of intervention will be tested by means of the net-benefit approach [38], and its cost-utility by using QALYs generated from EQ-5D.

Analysis will be carried out according to the principle of intention-to-treat. In case attrition should exceed the projected rate of 10%, additional per-protocol analysis will be performed.

### Data monitoring and safety

All trial data will be stored safely at the participating centres. The CC is responsible for the merging of the data from the centres and – after data cleaning – redistributing it to the centres for analysis.

Data Monitoring will be carried out by persons independent of trial conduct. Before start of data collection, each participating centre will name a person responsible for data entry and monitoring to the CC. The following monitoring strategy will be adhered to.

#### Monitoring of patient recruitment, data collection and application of the intervention

Study visits and regular telephone conferences will allow for a 100% check of essential trial parameters. In particular, the CC will see to that: (a) All informed consent forms which have been signed by patients are complete and safely stored at the participating centres; (b) Every participating patient meets inclusion criteria; (c) Both the intervention and assessment of outcome criteria, in each individual patient, are carried out according to the trial protocol and intervention manual. In addition, before start of recruitment, trial staff responsible for data collection and application of the intervention will be trained in the use of study instruments at the CC, also by external experts if applicable. As the study progresses, adherence to instrument and intervention manuals will be continuously monitored and training refreshed if necessary, especially in case of staff changes.

#### Monitoring of flow and quality of data

(a) At each centre, data will be entered via identical templates programmed by CC staff using *EpiData *, a reliable software for entering and documenting data allowing for e.g. the definition of out-of-range and outlier data as well as logic checks; (b) After data entry, centres will send copies of data files and completed questionnaires to the CC which will carry out: (i) Review: 10% of the data will be entered again by the CC and subsequently compared with the data in the files received from the participating centres. In case of major differences, proportion of the reviewed data will be increased; (ii) Query: In case of mismatch, the CC will contact the respective participating centre in order to correct data problems. Results of these queries will be communicated to the group.

Furthermore, a trial steering committee (TSC) was established in its function as an independent board of members to provide overall supervision and protection for the trial participants and the principal investigators. In view of the non-invasive intervention, the risk for participating patients is considered marginal. Should, for any unforeseeable reason, the intervention or data collection place a burden on a given participant and should at the same time he or she be unwilling to terminate participation, the research worker and/or intervention worker are required to report this to the TSC who will then decide on whether or not the participant will have to be excluded. Such instances will be documented thoroughly.

The advisory board, the TSC and Ulm University Hospital will be continuously informed of study progress including data quality issues.

### Ethics

Patient confidentiality will be strictly maintained, i.e. by no means will publications contain person-related information. However, the conduct of a longitudinal trial requires patient follow-up. Thus, study participants' personal data (e.g. names and addresses) will be recorded for the duration of the trial. Patient ID data will be stored safely and kept separate from the data set used for analyses.

In the course of recruitment, the research worker will devote special attention to providing the patients with detailed information on the trial so they have a sound basis on which to decide on informed consent. It will be made clear to the patient that he or she can withdraw consent at any time during the course of the study without any consequences. In case the patient is under legal custody, the custodian's consent does not suffice. All participating centres' ethics committees will have issued positive votes on NODPAM before the start of the study.

## Discussion

There is broad consensus that relapse prevention is one of the major aims of aftercare. However, the success of attempts to reduce high re-hospitalisation rates in people suffering from severe mental disorders has been limited so far. Insufficient discharge planning and follow-up is considered one of the main reasons for limited community tenure and unfavourable clinical outcomes. Only a small number of RCTs have been conducted to test this assumption.

Literature reviews conclude that specific interventions targeting the needs of high utilisers and subsequently preventing unnecessary high service use should be developed and evaluated as to their use for patients and clinicians [9,10,39].

Furthermore, taking into account the finding that health service costs make up 76% of the total costs, and that 59% of these are inpatient costs [14], any intervention that is effective in reducing the heavy inpatient bed use of heavy users should have a substantial effect on the total service costs [12,38]. Since service use patterns of high utilisers appear to depend on service system rather than on individual patient variables [8], it has been specifically recommended to identify gaps in current service provision [9], and – as outlined above – discharge from psychiatric hospital obviously can be considered such a gap, particularly in fragmented service systems such as the German one.

If this needs-oriented discharge planning and monitoring intervention proves to be successful in this RCT, a tool will be at hand (a) to harmonise fragmented service provision, i.e. improve collaboration of in- and outpatient services and continuity of care; (b) to improve community tenure, clinical impairment, and quality of life; and (c) to reduce unnecessary inpatient treatment costs.

## Footnotes

1 These were: (a) Randomisation strata: Originally planned strata were centre, primary diagnosis, and GAF score at admission. GAF score at admission was replaced by gender and illness duration. (b) Original exclusion criterion was only that subjects with a primary diagnosis of substance abuse will be excluded. (c) Originally the Helping Alliance Questionnaire (HAQ) was intended to be used for the assessment of the quality of the therapeutic relationship (changed to STAR); (e) Application of instruments to assess treatment satisfaction (ZUF-8 and ZUF-THERA) and satisfaction with discharge planning; (d) Specification of details on amount and kind of clinician remuneration.

2 The CSSRI-EU also serves to assess necessary information pertaining to SES (work, financial and living situation) and service utilisation beyond inpatient care. Furthermore, basic patient data will be available via the "BADO" which has been implemented at all participating centres.

## Competing interests

The authors declare that they have no competing interests.

## Authors' contributions

TB is principal and coordinating investigator of NODPAM. BP is responsible investigator in charge of trial management. The study was initiated by TB and BP who jointly wrote up and revised the proposal and study protocol in close collaboration with local PIs (WG, HF, HEK, TS). RM substantially contributed to the methods section, in particular to details of the randomisation. SS prepared the final draft for publication. All authors contributed to the design and continuing management of the study.

## Pre-publication history

The pre-publication history for this paper can be accessed here:


